# Exploring the Impact of Web-Based vs. In-Person Exercise Training on Benefits and Adherence in Substance Use Disorder Interventions: A Pilot Study

**DOI:** 10.3390/healthcare12060684

**Published:** 2024-03-19

**Authors:** Roberto Montón-Martínez, Juan Arturo Ballester-Ferrer, Sabina Baladzhaeva, Noemí Sempere-Ruiz, Antonio Casanova-Lizón, Alba Roldan, Diego Pastor, José Manuel Sarabia, Alejandro Javaloyes, Iván Peña-González, Manuel Moya-Ramón

**Affiliations:** 1 Sports Research Centre, Department of Sport Sciences, Miguel Hernández University of Elche, 03202 Elche, Spain; rmonton@umh.es (R.M.-M.); juan.ballesterf@umh.es (J.A.B.-F.); sabina.baladzhaeva@alu.umh.es (S.B.); nsempere@umh.es (N.S.-R.); acasanova@umh.es (A.C.-L.); dpastor@umh.es (D.P.); jsarabia@umh.es (J.M.S.); ajavaloyes@umh.es (A.J.); ipena@umh.es (I.P.-G.); mmoya@umh.es (M.M.-R.); 2 Alicante Institute for Health and Biomedical Research (ISABIAL), 03010 Alicante, Spain

**Keywords:** substance use disorders, adherence, mental health, quality of life, physical condition

## Abstract

Background: Substance use disorders pose unique challenges, affecting individuals physiologically and socially. This study addresses the fundamental question of how adherence to exercise programs impacts those with substance use disorders, examining both in-person and online interventions. Methods: A 12-week analysis involving 26 participants assessed physical fitness, strength, and quality of life. Participants were categorized into in-person and online exercise groups, with their adherence tracked through attendance and a dedicated app. Results: The in-person group exhibited higher adherence rates and significant improvements, in contrast to the challenges encountered by the online groups, particularly in substance use disorder cohorts. Statistical analyses highlighted these differences, emphasizing the pivotal role of the exercise program delivery format. Conclusions: This research advocates for hybrid models, blending professional supervision with online flexibility, recognizing the distinct challenges of substance use disorders. Insights from this study will contribute to shaping more effective, personalized interventions in the complex landscape of substance use disorders, offering guidance for advancing treatment strategies.

## 1. Introduction

According to the Diagnostic and Statistical Manual of Mental Disorders [1], substance use disorders are complex conditions that occur when the repeated use of a substance (e.g., alcohol, tobacco, and other legal and illegal substances) causes clinically significant psychophysical distress and impairment, including increased use over time, craving, tolerance, and social harm. This disrupted and continued use over a long period of time often leads to a wide spectrum of physical, psychological, and social consequences, which reduces and interrupts the participation of these people in recreational, social, or occupational activities, isolating them socially [2]. In fact, these are the same individuals who often suffer significantly from emotional and social loneliness [3].

The complexity of these chronic diseases requires multifaceted treatments. Within the variety of approaches, several reviews and meta-analysis studies have demonstrated that exercise may be employed for both the prevention and treatment of substance use addiction [4,5], in conjunction with traditional therapy. On the one hand, exercise programs are cost-effective, free of side effects, and confer multiple health benefits [6]. On the other hand, the positive impact of physical exercise has also been achieved in users with substance use disorders [7].

Despite their benefits, adherence to physical exercise programs among people with substance use disorders has been a challenge [8,9,10]. This lack of adherence prevents users from deriving the desired health benefits and leads to inconclusive results in research [11]. Previous research has attempted to identify the factors explaining these low adherence rates. Some studies have highlighted aspects that are more related to the individuals themselves, such as demographic aspects (e.g., age and gender), psychological factors (e.g., the severity of mental health symptoms and illness duration) [12,13], or the type of substance used [11]. Along the same lines, dropout rates were higher for cocaine, methamphetamines, and major stimulants and lower for alcohol, tobacco, and heroin [11].

A line of research in this area has addressed the issue by identifying the factors that encourage individuals to adhere to physical exercise routines [14]. Drawing from these insights, it has been proposed that users have certain preferences and motivations in terms of the content and structure of the exercise intervention (e.g., preferred location, social environment, frequency, or activity type) [15]. In terms of activity type, it is commonly observed that interventions are very diversified, and a wide range of activities are offered to ensure that participants find an activity that best suits their needs. According to a recent systematic review, these activities range from aerobic exercises (e.g., jogging, cycling, or walking) and mind–body activities (e.g., yoga and Tai chi, among others) to others like ball games [4]. Regarding structure, in mental health interventions, in-person programs have historically held greater significance. However, since the onset of the COVID-19 pandemic, online physical exercise programs have emerged as an indispensable resource for engaging with individuals with mental health issues. The flexibility and accessibility inherent to these online programs allow individuals to integrate tailored routines into their schedules and preferences [16]. The range of classes, spanning from yoga to high-intensity workouts, provides options catering to diverse skill levels and preferences. The virtual environment fosters a supportive community, mitigating isolation and enhancing emotional well-being [17]. Additionally, online interventions resolve the issues related to the activity’s cost and the time required to relocate to the facilities, while granting 24 h access to the activity’s content and interactions with the practitioner outside of time frames [18]. At the same time, learning when using digital technologies, such as wearables and internet, improves cognitive functioning and drives behavioral changes, treatment engagement, and abstinence, among others [19]. Thus, physical activity web-based programs are emerging as a part of mental health treatment for different populations like adults with depression, anxiety, or schizophrenia [20,21,22]. Unfortunately, online programs are almost nonexistent for adults with substance abuse. This is surprising, since the theory of the “habit” mechanism of exercise intervention in substance use disorder has already been demonstrated [23], which suggests that, through repetition and reinforcement, engaging in regular physical activity can replace addictive behaviors by forming new, healthier habits [24].

Therefore, the objectives of this pilot study are: (i) to design two different physical exercise programs—in-person and web-based—to observe the adherence of adults with substance abuse disorders, and (ii) to investigate the effectiveness of both programs in enhancing participants’ physical condition and mental health.

## 2. Materials and Methods

### 2.1. Participants

The total sample in the present study consisted of 26 individuals: those who were diagnosed with substance use disorder (SUD; n = 20) and healthy adults (HA; n = 6). Individuals with SUD were recruited from the rehabilitation center “Proyecto Hombre” in Alicante, Spain. The facility provides both inpatient and outpatient care. The subjects without SUD were recruited from the community using social media, flyers, and word-of-mouth. The subjects were classified into three exercise groups upon their recruitment, as described below. Inpatients were allocated to the in-person SUD group (n = 20). Conversely, both outpatients and subjects without an SUD diagnosis were organized into two distinct web-based exercise groups (online SUD group and online HA group, respectively). Descriptive data of the sample are provided in Table 1.

### 2.2. Experimental Design

The baseline assessments were spread over one week and included tests to evaluate cardiorespiratory fitness, strength, and quality of life (QoL). After the period of the baseline evaluations, each participant followed the same 12-week training program, but some did so in-person (in-person SUD group), while others did online (online SUD and online HA groups). Once the 12-week intervention was over, the participants were subjected to one week of final evaluations with the same assessment protocol as that at baseline.

#### Training Program

All groups followed the same training program. The only distinction was that, while the in-person groups engaged in coach-guided training sessions, the online training groups had access to identical pre-recorded training sessions through a mobile application (Selftraining UMH).

The training regimen consisted of three weekly sessions, each lasting 45–60 min. The total duration of the program was 12 weeks. All sessions were planned and supervised by two sports science professionals. Each session included a warm-up, main part, and cool-down. The warm-up phase comprised joint mobility exercises and dynamic movements that resembled the exercises included in the main part. A combination of strength and endurance exercises constituted the main part, with the intention of improving overall physical fitness, with a particular emphasis on muscular endurance and aerobic capacity. Each session was finalized with the cool-down. The progression of the load was performed following the four-phase approach previously described in Casanova et al. [25]. These phases were characterized as follows:

Phase 1 aimed to familiarize the participants with the training and teach proper techniques. Strength exercises such as squats, overhead presses, lunges, push-ups, and rows were performed, along with exercises targeting the core muscles, such as front planks, side planks, dynamic and static dorsal bridges, dead bugs, and bird dogs. Each exercise was performed for 15 repetitions, followed by a one-minute rest. During this rest period, core exercises or active planks were incorporated for 20 s, followed by 40 s of passive rest. Each exercise was repeated four times before moving on to the next, and approximately 4–5 exercises were performed. There was a two-minute rest between exercises.

Phase 2 introduced a higher intensity and a variety of exercises. It combined the strength exercises mentioned above with aerobic exercises like mountain climbers, skipping, jumping jacks, and boxing. Each exercise was performed for 30 s, followed by a 30-s rest. In each session, 10–12 exercises were performed in each set, with a total of two sets.

Phase 3 maintained the focus on combining aerobic and strength exercises, but the volume was increased. Three sets were performed instead of two, and the number of exercises per session was reduced to 8–10. The duration of exercises and rest intervals remained the same (30 s of work with 30 s of rest and a two-minute rest between sets).

For the last three weeks and during Phase 4, the characteristics of Level 3 were retained, but the duration of each repetition was increased to 40 s, and rest periods were reduced to 20 s. The two-minute rest between sets remained unchanged. The number of exercises and sets remained the same as in the previous level.

### 2.3. Evaluation

The assessed physical fitness variables, including aerobic capacity, upper limb strength, and lower limb strength, remained consistent across all study groups. However, it is noteworthy that specific tests for each cohort, i.e., adults with SUD or healthy adults, were chosen based on the distinctive characteristics of each population. This ensured a precise and appropriate measurement of the relevant physical parameters.

#### 2.3.1. Physical Fitness Test for SUD

- 6-min Walk Test (6MWT)

The 6-min Walk Test (6MWT) [26] was performed to evaluate aerobic capacity. The subjects were asked to walk for 6 min, covering the maximum distance possible in a designated area. The rectangle-shaped test area measured 5 yards (4.57 m) in width and 20 yards (18.28 m) in length, making a total of 50 yards covered in each lap. During the execution, the participants were warned twice about the remaining time, at 3- and 2-min marks before the test cessation.

- Curl Test (Upper-body strength)

The Curl Test [26] was employed to assess upper limb strength. For the test, each participant was seated on a chair with a dumbbell in their dominant hand. The participants were then asked to perform the maximum number of elbow flexions in 30 s. The dumbbell weighed five pounds (2.27 kg) for females and eight pounds (3.63 kg) for males.

- Chair Stand Test (lower-body strength)

The lower limb strength was evaluated using The Chair Stand Test (CST) [26]. For the test, the participants were required to stand up from a chair and sit back down as many times as possible in 30 s without using their arms. The participants were instructed to start from the seated position, and once the evaluator gave the start, the countdown would begin. Only properly executed repetitions were counted where participants maintained their arms crossed on the chest, extended their knees fully on the stand-up portion of the exercise, and sat back down completely.

#### 2.3.2. Quality of Life

The TECVASP test [27] was administered to evaluate the QoL in the participants with substance abuse disorder. The test was originally created for the Spanish-speaking community, but was later translated and validated into English as HRQOLDA (the Health-Related Quality of Life for Drug Abusers) [28]. TECVASP is a 22-item Likert-type instrument. For each item, the participants were asked to express to which degree they had experienced the emotions or episodes described in a given item for the past month. The scores ranged from 1 (not at all) to 5 (a lot). The higher the sum for all twenty-two items, the higher the QoL, and vice versa.

#### 2.3.3. Physical Fitness Test for HA

- Aerobic capacity

The participants performed an incremental test on a treadmill to measure the maximum oxygen uptake (VO_2_ max). The exchange of respiratory gases was measured using the Metalyzer 3B (Cortex GmbH, Leipzig, Germany) breath by breath. The participants were not allowed to drink or speak during the test and were asked to refrain from intense exercise 24 h earlier. The standard Bruce protocol [29] begins at 2.7 km/h at a 10% inclination and increases elevation by 2% and speed by 1.3 km/h every 3 min. After the test, there was 3 min of cool-down at 4 km/h and a 1% incline.

- Upper body strength

Upper body strength was evaluated using the Bench Press Test on the Smith Machine (Technogym S.p.A., Cesena, Italy). In brief, following a warm-up, the participants were asked to position themselves on a flat bench, with the bar placed on the chest and hands grasping the bar at a preferred width. Upon receiving a sign from the researcher, the participants were asked to perform a maximum number of repetitions in one minute. Correct execution included fully extending the elbows in the upward phase of the movement and letting the bar touch the chest in the downward phase. The load was fixed at 35 kg for men and 15 kg for women [30].

- Lower body strength

Lower body strength was assessed using the Squat test. A brief warm-up preceded the test. During the warm-up, the participants were advised on the proper squatting technique. The box was set up to ensure 90 degrees of knee flexion in each repetition. The participants started with the feet placed hip-width apart in front of the box, and once they received a sign from the researcher, the participants started the movement by flexing at the knees and hips and descended until they reached the box. From here, the participants were required to fully extend back to a starting position and perform a maximum number of repetitions for one minute [31].

#### 2.3.4. Adherence

For the in-person training, we tracked attendance to measure adherence to the training program. For online groups, adherence was monitored through the SelftrainingUMH mobile application. Before the program started, the participants in online groups received a unique username and password to access the app. This way, researchers recorded the number of sessions that participants in online groups performed throughout the intervention period. Over the course of the 12-week program, a total of 36 exercise sessions took place. The participants attended these sessions voluntarily in both in-person and online modalities.

### 2.4. Data Analysis

The test values were normalized using Z-scores to enable comparison between groups in each fitness category [32]. Prior to statistical analysis, homogeneity of variance was confirmed with Levene’s test.

To assess the mean differences in the fitness variables between the three experimental conditions following the intervention, repeated-measures ANOVA 2 × 3 (Time: pre–post x group: in-person SUD, online SUD, and online HA) with group as a between-subject factor was performed. For adherence and QoL, one-way ANOVA was performed to detect differences between the three experimental conditions, with group as a fixed factor. Due to the existence of significant differences between the groups at baseline in QoL, an ANCOVA was performed using the initial value of the test as a covariate. A post hoc analysis with Bonferroni adjustments was carried out to identify which groups differed from each other.

## 3. Results

### 3.1. Fitness

**Figure 1:**
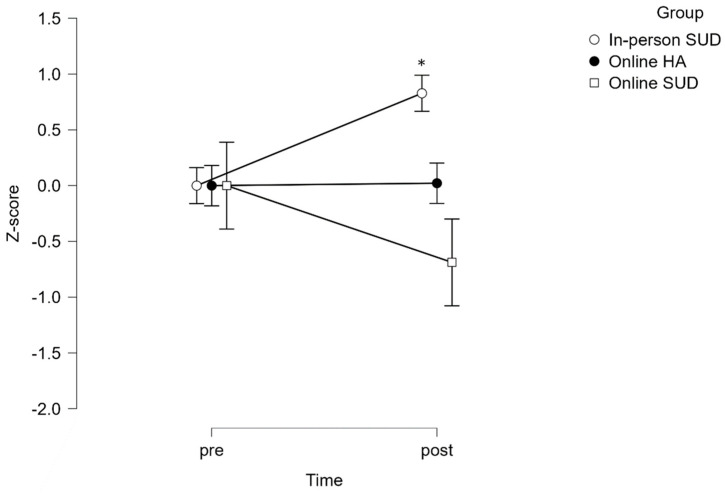
Group differences between pre and post for cardiorespiratory fitness. * Statistically significant differences from PRE (*p* < 0.05).

For cardiorespiratory fitness (Figure 1), the time x group interaction reached statistical significance (*p* = 0.01), but no effect of group (*p* = 0.385) was observed. Post hoc analysis adjustments detected statistically significant differences between the PRE and POST values of in-person SUD (*p* = 0.027), but no statistically significant differences were found in either online group.

**Figure 2:**
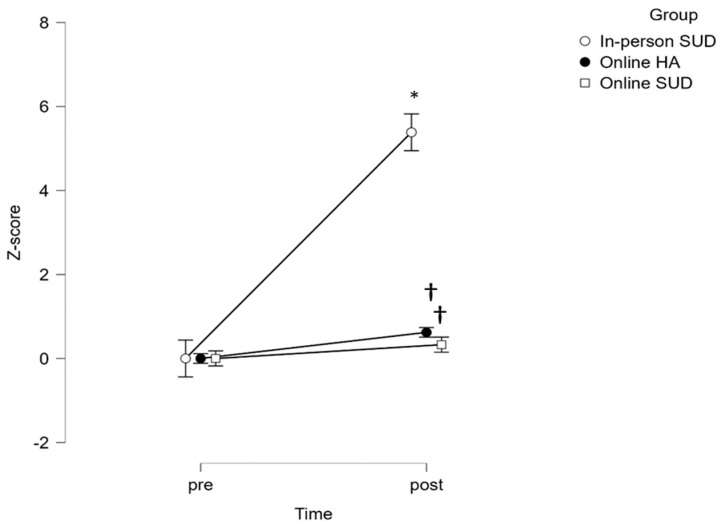
Group differences between pre and post for lower body strength. * Statistically significant differences from PRE (*p* < 0.05). † Statistically significant differences from in-person SUD (*p* < 0.05).

**Figure 3:**
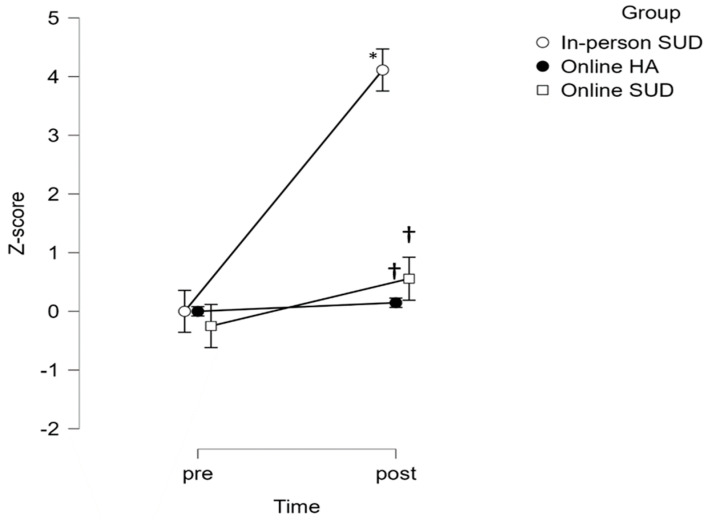
Group differences between pre and post for upper body strength. * Statistically significant differences from PRE (*p* < 0.05). † Statistically significant differences from post in-person SUD (*p* < 0.05).

For both lower- and upper-body strength (Figure 2 and Figure 3), the time x group interaction reached statistical significance (*p* < 0.0001), with the effect of group (*p* = 0.002 and *p* = 0.003, respectively). Post hoc analysis adjustments revealed statistically significant changes following the intervention in the in-person SUD group (*p* < 0.0001) and between groups in the post evaluation: in-person SUD vs. online SUD (*p* < 0.0001) and in-person SUD vs. online HA (*p* < 0.0001).

### 3.2. Adherence

Statistically significant differences were observed (*p* < 0.0001). The post hoc analysis demonstrated statistically significant differences between online HA and online SUD (*p* < 0.0001) and between in-person SUD and online SUD (*p* < 0.0001).

### 3.3. Quality of Life

**Figure 4:**
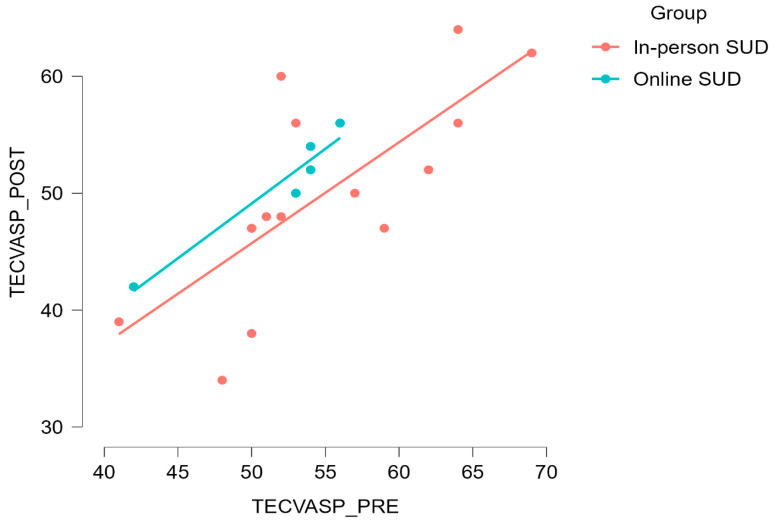
Group differences between pre and post for TECVASP scores.

Repeated-measures ANOVA revealed a significative decrease in the TECVASP score without differences between groups. However, baseline differences between groups were identified, warranting the use of ANCOVA. However, no further differences between groups were observed (Figure 4).

## 4. Discussion

The objectives of this study were twofold: to compare the adherence rates between web-based and supervised exercise programs for individuals with drug and alcohol addictions and to evaluate the effectiveness of both programs in improving physical fitness and mental health. As for the first objective, our results suggest that the delivery format of exercise programs significantly impacts user adherence and, consequently, the potential benefits they can derive.

According to our findings, the web-based format did not resonate well with the participants suffering from substance use disorders, while the opposite was true for the in-person program group, with higher adherence rates. Consistent with the existing literature, having a connection point to a center appears to be a key factor in achieving program adherence. Individuals who stay connected to a professional team and receive ongoing support and feedback are more likely to adhere to programs and experience better health-related benefits [33]. This was the key issue identified in the present study. Facilitating the activity in a structured way and with the added benefit of social interaction was beneficial in increasing adherence to the program [34]. However, as soon as the individual has completed the treatment period and left the center, in-person programs may be substituted by purely web-based models. Moreover, scientific evidence does support the online format for individuals with other mental health issues, such as depression and anxiety [35]. In this pilot study, however, it appeared that fully online intervention programs for individuals with substance use disorders may compromise their recruitment and adherence and, therefore, do not appear to be a feasible tool for this population. This is likely why the trend is to opt for in-person or hybrid formats for similar interventions. For instance, Krentzman et al. [36] conducted a hybrid study with outpatients battling alcohol use disorders, employing digitization solely for data collection. In that study, the exercise intervention itself took place at the rehabilitation center, resulting in satisfactory adherence rates among the participants.

Based on the results of this work, hybrid programs, combining the benefits of both types of delivery formats, may be the most interesting setup for increasing exercise adherence in individuals with substance use disorders. Nonetheless, the authors recommend further studies in hybrid formats for these individuals, considering various strategies recommended in the literature that may help to reduce dropout rates and enhance compliance. These strategies include regular monitoring by sports coaches, access to online forums for social support, and user-friendly web applications [16].

Concerning the second objective of the study, the observed enhancement in fitness levels among the participants in the in-person group aligns with previously documented findings [37,38,39,40]. Individuals with SUD often exhibit physical deterioration and diminished fitness compared to the general population [41]. Elevating fitness levels is crucial for drug-dependent patients to potentially prevent or alleviate various physical comorbidities [38,41]. The enhancements in QoL mirror outcomes reported by Muller and Clausen [42]. However, these notable fitness improvements were not observed in the online groups, both in the online SUD group due to insufficient adherence to the exercise program and in the online HA group, despite program completion. In the latter case, as indicated by Kraal et al. [43] in cardiac patients, this discrepancy may stem from failure to reach the target intensity during online training sessions, attributed to self-regulation of intensity.

Drug-dependent patients often experience severe bouts of anxiety and depression [44,45], as well as anhedonia [46,47]. The exercise program reported here led to a significant improvement in TECVASP test score, indicating an improvement in the perception of quality of life, but only in the in-person SUD group. In contrast, the online SUD group showed no significant changes in their test score, which aligns with their low adherence rate to the training program.

To conclude, the authors acknowledge the small sample size for this pilot study as a primary limitation, particularly for both online groups. Despite including participants of both sexes, the limited sample size poses challenges for meaningful gender-based comparisons. Moreover, addressing the difficulty in recruiting participants who have recently exited rehabilitation centers is crucial, particularly as it is challenging to obtain volunteers who are sedentary for exercise studies. This limitation affects the generalizability of these findings, emphasizing the need for future studies to explore innovative recruitment strategies. In upcoming research, it would be imperative to explore alternative avenues for recruiting sedentary individuals, considering motivational factors or potential incentives to enhance participation and representativeness.

Furthermore, it is crucial to highlight that the study did not account for the participants’ pre-existing physical activity habits, a factor identified by Zhang and Liu [23] as vital for the success of exercise-based intervention programs. The authors recommend promoting hybrid models in rehabilitation centers to cultivate optimal exercise habits in individuals before transitioning to purely web-based formats. This approach aligns with the emphasis on building long-term exercise habits, emphasizing the importance of a holistic strategy in designing effective rehabilitation programs.

## 5. Conclusions

In conclusion, this study addressed two primary objectives: comparing the adherence rates between web-based and supervised exercise programs for individuals with substance use disorders and evaluating the effectiveness of both programs in improving physical fitness and mental health. Our findings underscore the critical impact of the program delivery format on user adherence, with the in-person program demonstrating higher adherence levels than its web-based counterpart within the substance use disorder population. The incorporation of a connection point to a professional team emerged as a key factor contributing to program adherence, supporting the viability of hybrid models in the context of substance use disorder interventions.

While purely web-based interventions have shown success in general populations and other mental health conditions, our study highlights challenges in applying this format to individuals with substance use disorders, emphasizing the importance of ongoing support and feedback. Hybrid models present a pragmatic solution to enhance exercise adherence, but the long-term sustainability of these models may be compromised as individuals discontinue center attendance over time.

Regarding the improvement in fitness levels and quality of life, notable enhancements were observed in the in-person group, consistent with the existing literature on the positive impact of supervised exercise programs for individuals with substance use disorders. However, online groups, particularly the substance use disorder cohort, faced challenges in achieving similar benefits due to low adherence to the exercise program. This discrepancy emphasizes the need for tailored strategies to address the unique challenges associated with substance use disorders, such as regular monitoring, social support forums, and user-friendly applications.

Acknowledging the limitation of not considering the participants’ existing physical activity habits, our study suggests promoting hybrid models in rehabilitation centers to establish optimal exercise habits before transitioning to purely web-based formats. In addition, the constraints stemming from the small sample size utilized in our study may compromise the generalizability of our findings. As a pilot investigation, our results should be approached with caution, primarily serving as an indicative foundation for subsequent research on a broader scale. Future studies are encouraged to replicate and extend our work to validate the identified patterns and investigate the transferability of our findings across diverse populations. The incorporation of a larger and more heterogeneous sample would facilitate a more thorough evaluation of the efficacy of web-based versus supervised exercise interventions among individuals with substance use disorders, thereby elucidating the role of hybrid models in promoting exercise adherence and overall outcomes.

## Figures and Tables

**Table 1:** Descriptive data of the sample.

	In-Person SUD Group	Online SUD Group	Online HA
Men/women	10/5	4/1	3/3
Age (years)	47.5 ± 6.5	46.8 ± 8.5	38.0 ± 9.7
Weight (kg)	81.9 ± 13.0	84.0 ± 15.6	65.8 ± 13.7
Height (cm)	170.9 ± 7.8	172.5 ± 8.9	166.42 ± 7.4

Data are given as the mean ± standard deviation.

## Data Availability

The datasets generated from the current study are available from the corresponding author on reasonable request.
